# Supplementary material for: A Skin-Conformal, Stretchable, and Breathable Fiducial Marker Patch for Surgical Navigation Systems
Source: Micromachines (Basel). 2020 Feb 13;11(2):194. doi: 10.3390/mi11020194 (PMC7074652; doi:10.3390/mi11020194)
Supplement: Supplementary file 1 [file micromachines-11-00194-s001.pdf]

Supplementary Materials: A Skin-Conformal, Stretchable, and Breathable Fiducial Marker Patch for Surgical Navigation Systems

Sangkyu Lee, Duhwan Seong, Jiyong Yoon, Sungjun Lee, Hyoung Won Baac, Deukhee Lee and Donghee Son

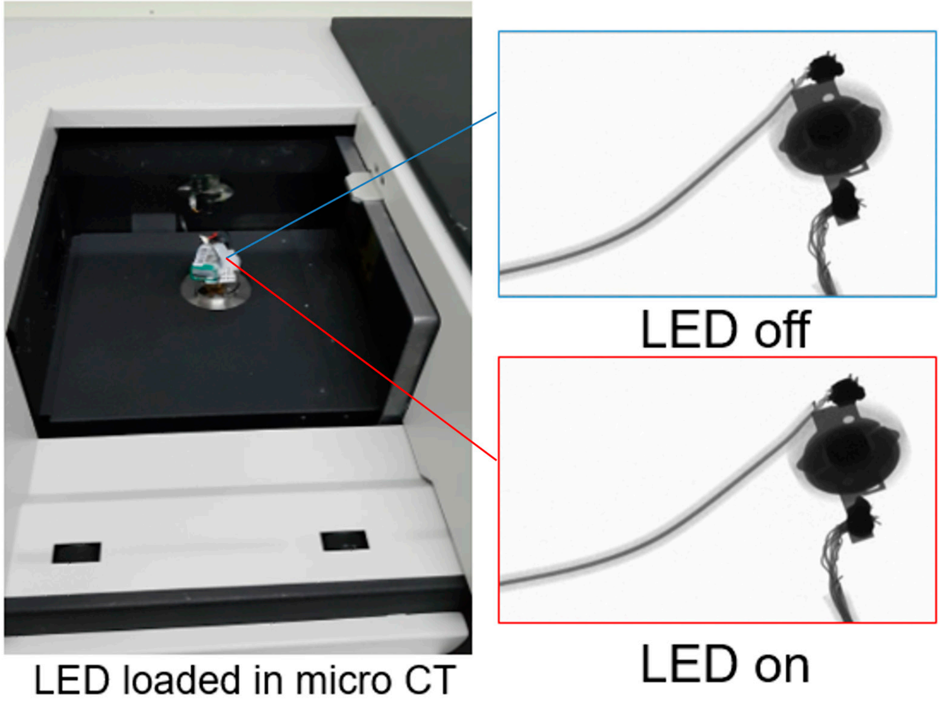

Figure S1. X-ray stability and recognition of the micro-IR LED with a micro-CT machine.
